# A case of severe bilateral empyema due to *Streptococcus pyogenes*

**DOI:** 10.1016/j.idcr.2023.e01848

**Published:** 2023-07-08

**Authors:** Tomoki Nishida, Takahisa Ohnishi, Takuya Kakutani, Nobuo Yamaguchi, Takayuki Kanemaru, Tomohiro Takenoue, Ryuta Fukai, Kazuto Inoue

**Affiliations:** a Department of General Thoracic Surgery, Shonan Kamakura General Hospital, 1370-1 Okamoto, Kamakura, Kanagawa Prefecture, Japan; b Department of General Surgery, Yamato Tokushukai Hospital, 4-4-12 Chuo, Yamato, Kanagawa Prefecture, Japan; c Department of Respiratory Medicine, Shonan Kamakura General Hospital, 1370-1 Okamoto, Kamakura, Kanagawa Prefecture, Japan

**Keywords:** Pleural empyema, *Streptococcus pyogenes*, Bilateral empyema

## Abstract

Bilateral empyema is a rare and severe condition and deciding on a treatment is quite difficult. Additionally, infections caused by group A *Streptococcus* (GAS [*Streptococcus pyogenes*]) are known to be invasive. We successfully treated without surgery a previously healthy 59-year-old woman with bilateral empyema due to GAS, with repeated drainages, antibiotics, and fibrinolytic therapy. To our knowledge, there have not been any published reports on cases of bilateral empyema due to GAS infection. In rare, severe cases of bilateral empyema caused by organisms such as GAS, physicians managing the condition should consider the overall condition of the patient.

## Introduction

Generally, acute empyema is a severe infection that is associated with pneumonia, and it has a high mortality rate [1]. It is usually unilateral; bilateral cases are rare and life-threatening condition [2]. Accordingly, the treatment of patients with bilateral empyema would be expected to be of increased difficulty. Furthermore, the safety of surgical procedures for the condition have not yet been established.

β-hemolytic Lancefield group A *Streptococcus* (GAS [*Streptococcus pyogenes*]) is a well-known causative pathogen of infections of the upper respiratory tract and skin. GAS infections occasionally lead to streptococcal toxic shock syndrome(STSS) [3], [4]. Although GAS is an uncommon pathogen involved in community-acquired pneumonia, the clinical course of a pneumonia caused by GAS is usually characterized by a sudden onset and a severe course. Moreover, GAS is rarely isolated from pus specimens [5], especially from adult patients and patients with bilateral involvement. Herein, we report a rare case of bilateral empyema due to GAS.

## Case report

A 59-year-old woman was admitted to our hospital with a 6-day history of fever and severe back pain. She did not have a significant past medical history. She was a nonsmoker, did not drink alcohol, and was not taking medication for other medical conditions. She worked as a child-care provider, and usually practiced karate or tai chi.

**Fig. 1:**
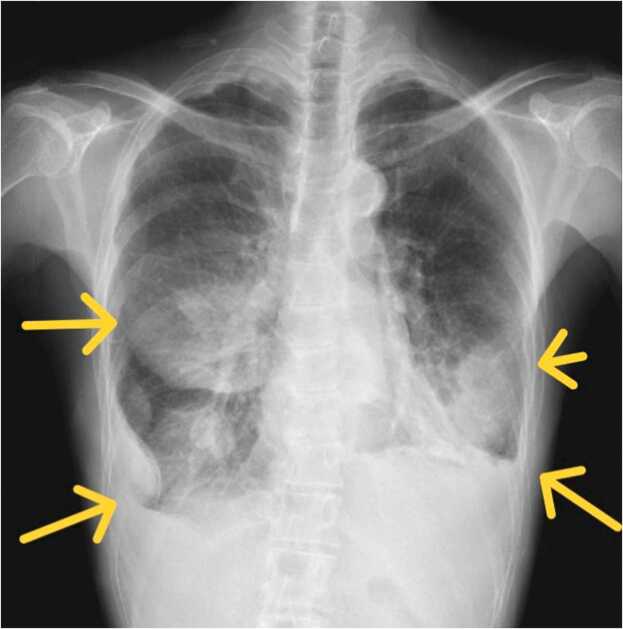
Chest radiograph on admission. That revealed the lungs were hypolucent bilaterally, with dullness of the costophrenic angle (yellow arrows).

**Fig. 2:**
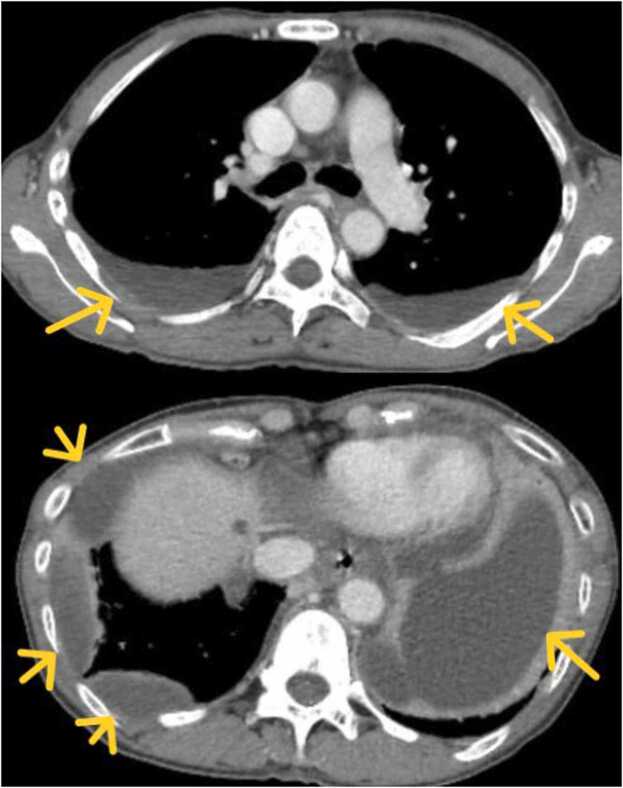
Computed tomography scans(CT) on admission. That revealed bilateral posterior and basal pleural effusions (yellow arrows).

Upon admission to the emergency room, the patient was afebrile with an oxygen saturation of 94% on room air concomitant with dyspnea on exertion. Her blood pressure was 114/63 mm Hg and her heart rate was 128 beats/min. Her leukocyte count was 36,800/μL (reference range, 3300–8600/μL); C-reactive protein concentration was 27.4 mg/dL (reference range, 0.00–0.14 mg/dL); and levels of serum total protein, 5.6 g/dL (reference range, 6.6–8.1 g/dL); albumin, 2.3 g/dL (reference range, 4.1–5.1 g/dL); ferritin, 653.6 ng/mL (reference range, 12–60 ng/mL); procalcitonin, 5.86 ng/mL (reference range, ≤0.05 ng/mL); and d-dimer, 15.1 μg/mL (reference range, ≤1.0 μg/mL). Chest radiography revealed bilateral consolidation and dullness of the both costophrenic angle(Fig. 1), and chest computed tomography(CT) showed bilateral pleural effusions, which were located in the posterior and basal spaces (Fig. 2).

**Fig. 3:**
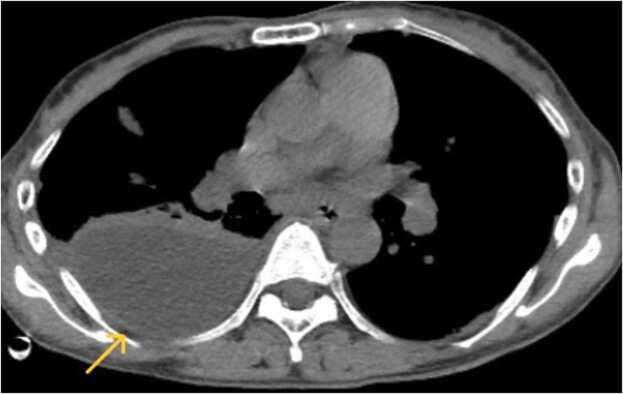
Follow-up CT on day six after hospitalization. That revealed residual pleural effusion in the right posterior space (yellow arrow), which had increased.

The patient was considered to be preseptic as a result of bilateral empyema, and bilateral chest tubes were inserted into the pleural space. Pleural effusate from both chest tubes appeared to be cloudy, yellowish pus. Urokinase fibrinolytic therapy (60,000 units per day) was administered intrapleurally through the chest tubes for three days. On day six after hospitalization, bacterial cultures of blood and pleural effusate specimens were positive for *S. Pyogenes*. Follow-up CT showed residual pleural effusion in the right posterior space (Fig. 3). An additional drainage tube was inserted to right pleural cavity, and urokinase fibrinolytic therapy was performed for another three days.

**Fig. 4:**
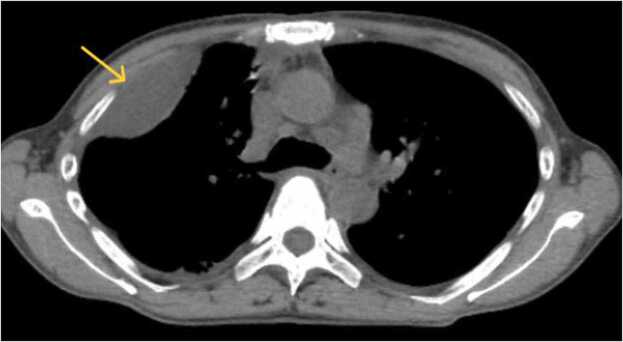
Follow-up CT on day fifteen after hospitalization. That revealed recurrent right pleural effusion in the right anterior space (yellow arrow).

**Fig. 5:**
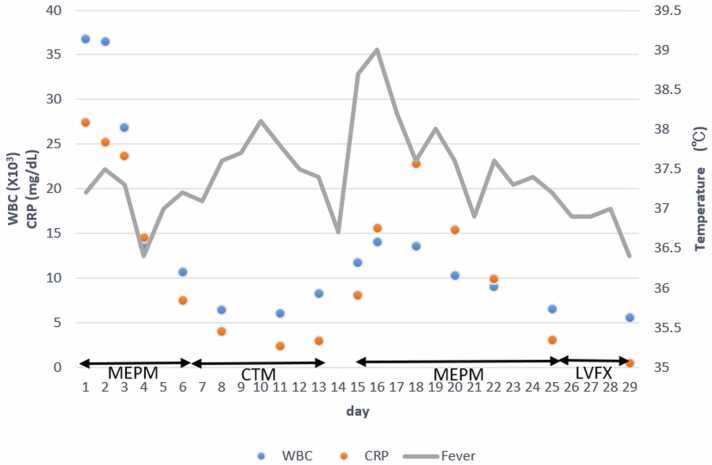
Clinical course of the patient. Change over time of white blood cell (WBC) counts, C-reactive protein (CRP) levels, and temperatures are shown. The following antibiotics were administered: MEPM, meropenem; CTM, cefotiam; LVFX, levofloxacin.

With subsequent gradual decrease in the residual pleural effusion and improvement in the patient’s laboratory indices, all the drainage tubes were removed on day 13 after admission. However, two days later, the patient developed a high fever and her laboratory indices worsened. A CT scan showed recurrent right pleural effusion in the anterior space (Fig. 4). Because it was a unilocular cavity, we decided that it could only be relieved by drainage without surgery, so a more minimally invasive option was chosen. A new drainage tube was inserted into the right pleural cavity, with subsequent removal five days later. The patient’s clinical course, including temperature, laboratory data, and antibiotic therapy is shown in Fig. 5. The patient was discharged on day 29 after admission. As for the use of antibiotics, we were de-escalated once after detecting sensitivity. However, since it worsened again, it was decided to return to broad-spectrum antibiotics and continue. Although penicillin can be used for GAS generally, we chose such antibiotics due to the unfamiliarity with treatment for GAS.

We subsequently received new clinical information on the patient from another hospital, where she had been seen for irregular bleeding seven days before admission to our hospital. A culture specimen from her vagina was positive for *S. pyogenes*, which was the same bacteria causing her empyema. We suspected that the source of her infection might have been a child where she worked in daycare.

## Discussion

The course of acute empyema is generally classified into three phases, the first is the exudative, the second is the fibrinopurulent, and the third is the organizing phase [1]. The American Association for Thoracic Surgery consensus guidelines have recommended video-assisted thoracoscopic surgery for acute empyema, especially for patients in the exudative and fibrinopurulent stages [6]. Molnar also reported that decortication via video-assisted thoracic surgery has shown high success rates, ranging from 68% to 93% [7]. The number of elderly patients with empyema due to aspiration pneumonia, which includes bedridden patients, has been increasing, and the operability of such patients has often been controversial. We reported the results from a retrospective study of patients with empyema in our institution and concluded that patients within a performance status (PS) of 2 and an American Society of Anesthesiologists physical status classification of Ⅱ were able to undergo surgical procedures for empyema without problems [8].

However, bilateral empyema is quite rare, and there are some difficulties associated with the surgical approach. First, the patients are difficult to move because of the severe pain caused by bilateral drainage tubes. Thus, they tend to develop more postoperative complications than usual if surgery is performed. Second, one-lung ventilation during surgery might be difficult to perform, which results in difficulties performing video-assisted thoracic surgery. Because poor oxygenation would prevent the use of general anesthesia with different lung ventilation, we focused on treating the pneumonia preoperatively[9].

According to the American College of Chest Physicians Parapneumonic Effusions Panel, the estimated risk for a poor outcome is determined by the anatomy of the pleural space and the bacterial and chemical composition of the pleural fluid[10]. Based on our patient’s chest CT findings and clinical course, we believed that it was highly probable that our patient’s condition could be improved by tube drainage only. Therefore, we decided not to perform surgery, unless her infection could not be controlled by tube drainage. We anticipated that at most, we might have to perform surgery as a secondary therapy for controlling the infection in her right pleural space.

GAS infections have often been accompanied by sepsis, and the manifestations of infections by GAS are difficult to treat easily. The severity of bilateral empyema due to GAS has been categorized as Class ⅡA, according to a classification of group A streptococcal infections[11]. The published literature provides some information on the mechanisms involved in GAS infections. Streptococcal degrading enzymes such as the IL-8 cleaving peptidase (ScpC) and the C5a peptidase (ScpA) have been found to weaken the infiltration of neutrophils [12]. GAS strains also express many bacterial virulence factors, including the M protein, and streptolysins and streptokinase. Of these factors, the M protein, which is encoded by the *emm* gene, is a major bacterial virulence factor that is resistant to phagocytosis[13].

GAS can colonize humans asymptomatically, but also is a frequent cause of pharyngeal and the skin infections. Therefore, the origins of GAS infections are often the upper respiratory tract or skin that has been damaged by trauma or surgery. GAS has caused invasive infections, including bacteremia, pneumonia, and STSS, which all have high mortality rates. There have been rare case reports on the occurrence of primary peritonitis in healthy women that were caused by GAS. It was thought that the infection might have spread from the vagina through the genitourinary tract[14]. We presumed that the origin of our patient’s GAS infection was the vagina, and that the GAS organisms spread hematogenously to both pleural spaces. We found our hypothesis to be very interesting.

GAS infections are more common in pediatric than in adult patients. The onset and progression of GAS infections are so rapid that prompt antibiotics therapy and early surgical debridement of infected tissue are essential for the treatment of pediatric infections [15]. Although some cases of pediatric empyema due to GAS have been reported, there have been few reports on adult cases of empyema due to GAS. Sakai et al. reported on a case of pleural empyema and STSS due to GAS in a healthy man that we found valuable, despite the fact that it was unilateral empyema [16]. Asai et al. reported an adult case of acute empyema due to GAS following an influenza A infection [17], and there have also been similar pediatric case reports. Robinson mentioned that the influenza A virus inhibits bacterial-induced IL-1β production and impairs host defense against bacterial infection [18]. We were unable to find any published case reports on bilateral empyema caused by GAS. We recommend that acute empyema caused by GAS should be managed carefully with focus on the condition of the patient’s lungs and severity of infection.

## Conclusion

We treated a quite rare case of bilateral empyema due to GAS, and to our best knowledge, this is the first report of such a case. The clinical management of this condition was very difficult. We hope that with further experience with similar cases, successful methods of treatment will emerge.

## Ethical approval

This was not a clinical research report, but a case report. We do not think that ethical approval is needed.

## Consent

The patient gave her informed consent before this article was written.

## Funding

This research did not receive any specific grant from funding agencies in the public, commercial, or not-for-profit sectors.

## CRediT authorship contribution statement

TO, TK, TT and KI were involved in the clinical care of the patients. TN wrote the initial draft. All authors read, revised, and approved the final manuscript.

## Declaration of Competing Interest

We do not have any conflicts of interest associated with this case report.

## Data Availability

Not applicable.
